# Hypoglycemia, Vascular Disease and Cognitive Dysfunction in Diabetes: Insights from Text Mining-Based Reconstruction and Bioinformatics Analysis of the Gene Networks

**DOI:** 10.3390/ijms222212419

**Published:** 2021-11-17

**Authors:** Olga V. Saik, Vadim V. Klimontov

**Affiliations:** 1 Laboratory of Endocrinology, Research Institute of Clinical and Experimental Lymphology—Branch of the Institute of Cytology and Genetics, Siberian Branch of Russian Academy of Sciences (RICEL—Branch of ICG SB RAS), 630060 Novosibirsk, Russia; saik@bionet.nsc.ru; 2 Laboratory of Computer Proteomics, Federal Research Center Institute of Cytology and Genetics, Siberian Branch of the Russian Academy of Sciences (ICG SB RAS), 630090 Novosibirsk, Russia

**Keywords:** hypoglycemia, diabetes, cardiovascular disease, diabetic retinopathy, diabetic nephropathy, cognitive dysfunction, Alzheimer’s disease, diabetic neuropathy, gene networks, ANDsystem

## Abstract

Hypoglycemia has been recognized as a risk factor for diabetic vascular complications and cognitive decline, but the molecular mechanisms of the effect of hypoglycemia on target organs are not fully understood. In this work, gene networks of hypoglycemia and cardiovascular disease, diabetic retinopathy, diabetic nephropathy, diabetic neuropathy, cognitive decline, and Alzheimer’s disease were reconstructed using ANDSystem, a text-mining-based tool. The gene network of hypoglycemia included 141 genes and 2467 interactions. Enrichment analysis of Gene Ontology (GO) biological processes showed that the regulation of insulin secretion, glucose homeostasis, apoptosis, nitric oxide biosynthesis, and cell signaling are significantly enriched for hypoglycemia. Among the network hubs, *INS, IL6, LEP, TNF, IL1B, EGFR*, and *FOS* had the highest betweenness centrality, while *GPR142, MBOAT4, SLC5A4, IGFBP6, PPY, G6PC1, SLC2A2, GYS2, GCGR*, and *AQP7* demonstrated the highest cross-talk specificity. Hypoglycemia-related genes were overrepresented in the gene networks of diabetic complications and comorbidity; moreover, 14 genes were mutual for all studied disorders. Eleven GO biological processes (glucose homeostasis, nitric oxide biosynthesis, smooth muscle cell proliferation, ERK1 and ERK2 cascade, etc.) were overrepresented in all reconstructed networks. The obtained results expand our understanding of the molecular mechanisms underlying the deteriorating effects of hypoglycemia in diabetes-associated vascular disease and cognitive dysfunction.

## 1. Introduction

Hypoglycemia is a life-threatening complication and a barrier to achieving good glycemic control in patients with diabetes [1]. The long-term consequences of hypoglycemia include cardiovascular events, as well as cognitive and psychological problems [2]. In both type 1 and type 2 diabetes, self-reported episodes of severe hypoglycemia are related to increased risk of death [3]. Large prospective clinical studies have documented the association between severe diabetes-related hypoglycemia and major adverse cardiovascular events: cardiovascular and all-cause mortality [4,5,6]. In diabetes, the link between severe hypoglycemia and cardiovascular events is time-dependent and bidirectional: this means increased cardiovascular risk after severe hypoglycemia, as well as greater risk of severe hypoglycemia after a cardiovascular event [7]. Recent studies indicate a promoting role of hypoglycemia in the progression of microvascular diabetic complications, including retinopathy [8,9] and chronic kidney disease [10]. Decreased kidney function, in turn, increases the risk of hypoglycemia [11].

In individuals with diabetes, severe hypoglycemia is an established risk factor for cognitive decline and dementia [12,13,14]. Recurrent symptomatic or asymptomatic hypoglycemia has been suggested to induce sub-clinical brain damage and permanent cognitive dysfunction [15]. Recent clinical observations suggest that hypoglycemia is a risk factor for both vascular dementia and dementia due to Alzheimer’s disease (AD) in elderly patients with type 2 diabetes [16]. These data are consistent with the results of experimental studies indicating that glucose deprivation triggers tau pathology and synaptic dysfunction in the brain, the hallmarks of AD [17,18]. It should be noted that type 2 diabetes can also increase the risk of AD [14].

It is well known that a glucose-deprived condition triggers a cascade of adaptive and pathophysiological events in the cardiovascular and nervous systems. Cardiovascular effects of hypoglycemia include increase in cardiac work load and potential attenuation of myocardial perfusion, potentially arrhythmogenic electrophysiological changes, induction of a prothrombotic state, and release of inflammatory mediators [19]. An episode of hypoglycemia induces an adaptive counter-regulatory response, which involves enhanced glucagon, epinephrine, cortisol and growth hormone secretion; the suppression of insulin release; and the modulation of the autonomic nervous system. Recurrent or chronic hypoglycemia induces multiple shifts in the brain’s metabolism, including glycogen mobilization; the utilization of alternate sources of energy, such as lactate and ketones; changes in glucose uptake; and changes in cellular respiration [20]. However, the molecular mechanisms of the effects of hypoglycemia in the target organs are not fully understood.

Artificial intelligence and bioinformatics open up new possibilities for systems analysis of molecular events in human diseases. Text-mining is a field in artificial intelligence that aims to extract information from collections of text documents based on machine learning and natural language processing techniques. Text-mining is considered a useful tool for integrative biomedical research involving genes, proteins and phenotypes [21]. In this study we applied ANDSystem (ICG SB RAS, Novosibirsk, Russia), a bioinformatics tool that builds molecular (gene) networks by text-mining of PubMed/Medline indexed publications [22,23,24], for reconstruction and analysis of gene networks of hypoglycemia and diabetes-related conditions for which hypoglycemia may be a risk factor.

The main goal of the ANDSystem is to allow the generation of new hypotheses related to understanding of the molecular mechanisms of complex biological processes by reconstruction and analysis of associative molecular (gene) networks where biological objects are presented as nodes, and interactions between them are presented as edges. For that purpose, a high-throughput technology of automatic knowledge extraction from texts of scientific publications is utilized. For the first step, the text-mining approach performs automated recognition of the names of biological entities in texts. For the second step, it reveals interactions between biological objects using more than 3000 specific semantic templates. The information extracted by the text-mining is stored in the huge ANDCell knowledge base which is updated annually. Information from the ANDCell knowledge base could be queried by users through the ANDVisio client module. The ANDVisio supports network visualization and analysis. For example, ANDVisio functions allow to calculate the connectivity and the centrality coefficients of nodes [22,23,24]. Previously, the ANDSystem was applied successfully to analyze the molecular basis of a number of human diseases and comorbidity [25,26,27,28].

One of the well-established ways to find relations between gene sets obtained in the research and the studied conditions (biological processes, diseases, phenotypes, etc.) is the gene set enrichment analysis. As a result of applying this method, it is possible to identify sets of genes for which the frequency of occurrence in the analyzed set, associated with the target condition, is significantly different from the background frequency (for example, the frequency in the entire genome). Such sets of genes are called overrepresented (if the frequency is higher than the background) or underrepresented (if the frequency is below the background). The hypergeometric distribution is commonly used as a statistical model to assess the significance of enrichment. The examples of web tools that perform the gene set enrichment analysis are DAVID [29] and TopAnat function of Bgee [30]. DAVID is aimed to perform comprehensive functional annotation for revealing the biological meaning of a large list of tested genes. It is in particular able to identify enriched Gene Ontology terms [29]. Bgee is a database containing information on gene expression patterns in different tissues and cells. Its TopAnat function allows to find enrichment of anatomical terms related to genes by expression patterns [30]. The gene set enrichment analysis was performed in this work to reveal interconnections between studied genes and hypoglycemia, vascular disease and cognitive dysfunction in diabetes.

Despite the obvious clinical importance of the issue, a gene network of hypoglycemia has not yet been analyzed. The comparative analysis of a network of hypoglycemia and diabetes-related diseases has not been performed also. Therefore, in this study, we reconstructed and matched the gene networks of hypoglycemia, cardiovascular disease, microvascular diabetes complications, cognitive dysfunction and AD with the use of ANDSystem to identify the principal molecules and processes that can mediate the effects of hypoglycemia on the target organs in diabetes.

## 2. Results and Discussion

### 2.1. Gene Network of Hypoglycemia

**Figure 1:**
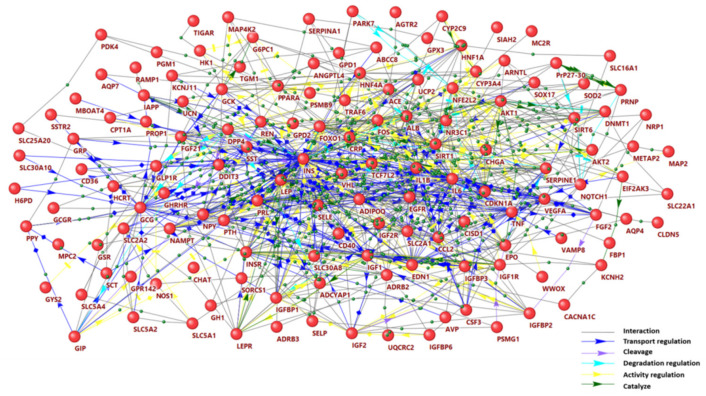
Molecular network of hypoglycemia visualized with the ANDSystem.

In our previous work [28], we have reconstructed a gene network associated with hypoglycemia in individuals with diabetes. This network included 128 genes/proteins and 2467 interactions. As the ANDSystem (ICG SB RAS, Novosibirsk, Russia) [22,23,24] was updated in 2021, the gene network related to hypoglycemia (Figure 1) has been expanded to include 141 genes/proteins and 5525 interactions (Table S1).

The network of hypoglycemia consisted of molecules with different structures and functions. It included insulin and other hormones, cytokines and growth factors, enzymes, transporters, transcription factors, neuropeptides, structural and binding proteins, and microRNAs (Table 1).

Expectedly, genes encoding hormones that regulate glucose metabolism including insulin (*INS*), glucagon (*GCG*), glucagon-like peptide 1 (*GCG),* glucose-dependent insulinotropic polypeptide *(GIP*), islet amyloid polypeptide (*IAPP*), growth hormone (*GH1*), and some hormonal receptors (*INSR, GLP1R, ADRB2, ADRB3*) turned out to be the central hubs of this network. Among identified hormones, insulin plays a key role as an inducer of hypoglycemia, glucagon and growth hormone are involved in the response to hypoglycemia, and other hormones act as modulators of insulin secretion or sensitivity. Alternatively, hypoglycemia itself may affect the secretion of a number of these regulators [31,32]. Some of the identified transcription factors (*HNF1A, HNF4A,* and *TCF7L2*) are essential for glucose homeostasis. A group of neuropeptides included modulators of the neuroendocrine system, such as adenylate cyclase-activating polypeptide 1(*ADCYAP1*), neuromedin C (*GRP),* and chromogranin A (*CHGA*), and some regulators of appetite and food intake, namely, hypocretin neuropeptide precursor (*HCRT),* neuropeptide Y *(NPY),* pancreatic polypeptide Y *(PPY),* and urocortin (*UCN*) participated in the network.

Two microRNAs genes (*MIR155* and *MIR410*) identified in the networks were both involved in glucose metabolism. Specifically, in mice, global overexpression of miR155 resulted in hypoglycemia, improved glucose tolerance and enhanced insulin sensitivity of peripheral tissues [33]. MiR-410 enhanced glucose-stimulated insulin secretion in vitro [34]. It is also involved in the brain response to oxygen-glucose deprivation [35].

The nature of the links in the network is shown in Table 2. It was found that 43 genes were up-regulated and 17 genes were down-regulated by hypoglycemia. In turn, the products of 16 genes were able to induce hypoglycemia, and 22 genes were described to have a protective effect against hypoglycemia or involved in the counterregulatory response. The SNPs associated with hypoglycemia were observed in 34 genes.

**Figure 2:**
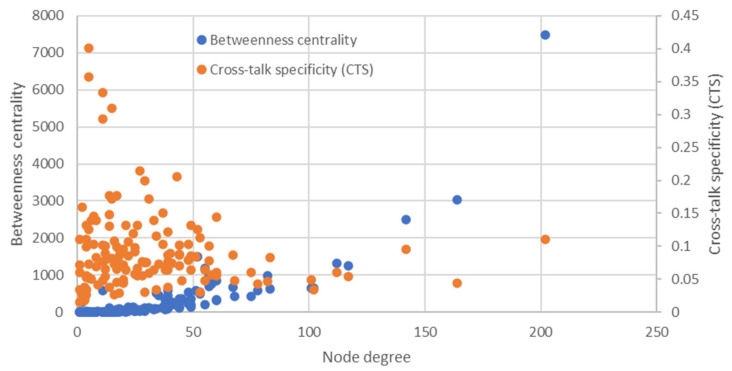
The distribution of node connectivity, betweenness centrality and cross-talk specificity (CTS) values in the hypoglycemia network.

The distribution of the number of gene connections in the network turned out to be exponential (Figure 2, Table S2). Only seven genes (*INS, IL6, LEP, TNF, IL1B, EGFR,* and *FOS*) showed over 100 connections with other elements of the network. All these junction genes were present among the top 15 genes with the highest betweenness centrality (Figure 2, Table S3), which suggests their key role in the hypoglycemia pathophysiology. The proteins encoded by these genes regulate glucose and lipid metabolism (*INS, LEP*), cell proliferation, differentiation and survival (*INS, LEP, EGFR, FOS*), inflammatory pathways (*IL1B, IL6, TNF,* *FOS*), angiogenesis (*IL1B, IL6, LEP, TNF, EGFR, FOS*), and other processes.

Ten genes (*GPR142, MBOAT4, SLC5A4, IGFBP6, PPY, G6PC1, SLC2A2, GYS2, GCGR* and *AQP7)* demonstrated the highest cross-talk specificity values (Figure 2, Table S3). These genes had a relatively large number of links in the considered gene network having a small number of links in the global human gene network. Among the products, islet-enriched G protein-coupled receptor (*GPR142*) was discussed as a potential target for the treatment of type 2 diabetes as it stimulates glucose-dependent insulin secretion [36]. Overproduction of insulin-like growth factor-binding protein 6 (*IGFBP6*) was reported to be a marker of non-islet tumor-induced hypoglycemia [37]. Mutations in the genes of glucose-6-phosphatase (*G6PC1),* glycogen synthase 2 (*GYS2*) and aquaporin 7 (*AQP7*) may cause fasting hypoglycemia due to impaired liver metabolism [38,39]. Glucagon receptor (*GCGR*), pancreatic polypeptide Y (*PPY*), glucose transporter 2 (*GLUT2, SLC2A2*) and ghrelin o-acyltransferase (*MBOAT4*) could be involved in the counterregulatory response to hypoglycemia [40,41,42,43].

An analysis performed with the DAVID web-tool [29] identified insulin secretion, glucose homeostasis, up-regulation of gene transcription, regulation of neuron death, apoptosis and nitric oxide biosynthesis among the most overrepresented Gene Ontology (GO) biological processes (Table S4, Table 3).

At the next step, we performed the enrichment analysis of anatomical structures mapped to genes by expression patterns by Bgee web-tool [30]. The central nervous system, connective tissue, muscles, cardiovascular system, gastrointestinal tract, female reproductive system, abdominal adipose tissue, kidney, and pancreas turned out to be the most overrepresented entities where the greatest number of hypoglycemia-related genes expresses (Table S5).

The obtained results clearly indicate that hypoglycemia can regulate a lot of hub genes affecting the key biological processes in the targeted organs.

### 2.2. Comparative Analysis of the Gene Networks of Hypoglycemia and Diabetic Vascular Disease

Taking into account the clinical association between hypoglycemia and vascular disease in diabetes, we matched the gene network of hypoglycemia with the gene networks of diabetic macrovascular and microvascular complications.

With the instruments of the ANDSystem, we have identified 494 genes/proteins associated with cardiovascular disease, of which 47 were also present in the gene network of hypoglycemia (Table S6). In addition, genes related to hypoglycemia were significantly overrepresented in the network of cardiovascular disease (*p*-value 10^−39^). The network of diabetic retinopathy contained 424 genes/proteins, fifty of them were also present in the network of hypoglycemia (Table S7). The network of diabetic nephropathy consisted of 685 genes/proteins; among them, 62 molecules shared with the hypoglycemia network (Table S8). One hundred and thirty genes/proteins made up the network of diabetic neuropathy; among them, 22 were found in the gene network of hypoglycemia (Table S9). In all networks of microvascular complications, the genes related to hypoglycemia were significantly overrepresented, with *p*-values 10^−45^, 10^−53^ and 10^−23^ respectively.

According to the AmiGO2 database (http://geneontology.org/), 104 human genes are associated with microvascular endothelial cells (Table S10). Of these, 15 were present in the hypoglycemic gene network, including CD36 molecule (*CD36*), claudin 5 (*CLDN5*), epidermal growth factor receptor (*EGFR*), fibroblast growth factor 2 (*FGF2*), insulin receptor (*INSR*), leptin (*LEP*), leptin receptor (*LEPR*), notch 1 (*NOTCH1*), solute carrier family 16 member 1 (*SLC16A1*), solute carrier family 22 member 1 (*SLC22A1*), solute carrier family 2 member 1 (*SLC2A1*), solute carrier family 5 member 1 (*SLC5A1*), tumor necrosis factor (*TNF*), TNF receptor-associated factor 6 (*TRAF6*), and vascular endothelial growth factor A (*VEGFA*). The chance of finding so many genes for random reasons is extremely low (*p*-value 10^−15^).

**Figure 3:**
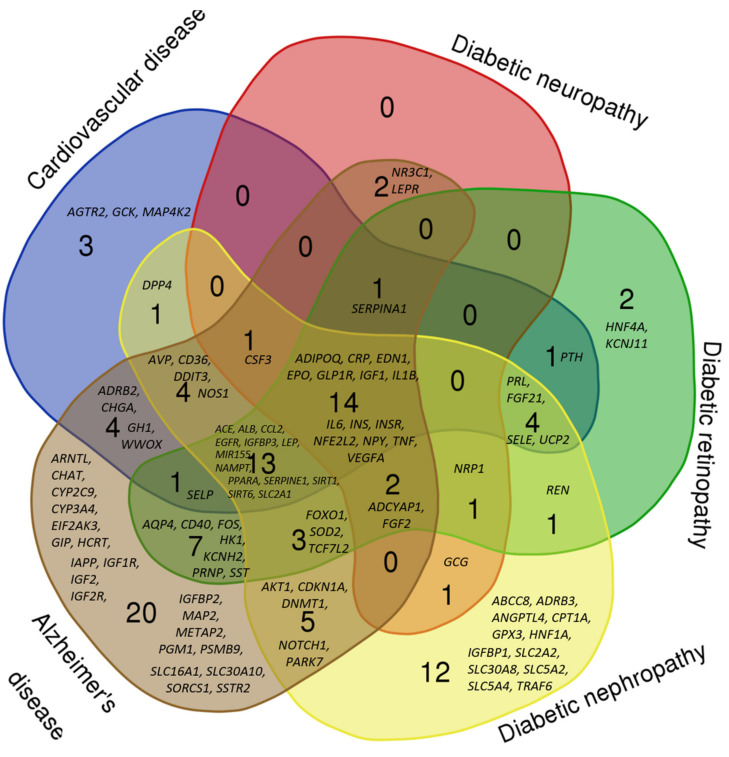
Venn diagram of intersection of gene sets associated simultaneously with hypoglycemia and cardiovascular disease, diabetic neuropathy, diabetic retinopathy, diabetic nephropathy, and Alzheimer’s disease.

Figure 3 represents a Venn diagram showing the intersection of the gene lists of the networks of hypoglycemia, cardiovascular disease, diabetic retinopathy, diabetic nephropathy, and diabetic neuropathy. It turned out that 14 genes were mutual for all analyzed gene networks. The products of these genes encode insulin (*INS*) and insulin receptor (*INSR*), endothelin-1 (*EDN1*), erythropoietin (*EPO*), adiponectin (*ADIPOQ*), interleukin-1β (*IL1B*), interleukin-6 (*IL6*), tumor necrosis factor α (*TNF*), glucagon-like peptide-1 receptor (*GLP1R*), insulin-like growth factor-1 (*IGF1*), vascular endothelial growth factor A (*VEGFA*), C-reactive protein (*CRP*), nuclear factor, erythroid 2 like 2 (*NFE2L2*) and neuropeptide Y (*NPY*). A wide range of biological activities of these molecules is consistent with the concept of their key role in the pathophysiology of diabetic vascular complications [44,45,46,47,48,49,50,51,52,53,54,55,56].

In addition, the genes of neuropilin 1 (*NRP1*), adenylate cyclase-activating polypeptide 1 (*ADCYAP1*) and fibroblast growth factor 2 (*FGF2*) were mutual for the networks of hypoglycemia and all microvascular complications. Neuropilin-1 is a membrane-bound receptor for vascular endothelial growth factor and semaphorin family members, it is important for angiogenesis, axon guidance, cell survival, migration, and invasion. The role of neuropilin-1 in diabetic complications is discussed [57,58]. Adenylate cyclase-activating polypeptide 1, the product of *ADCYAP1* gene, is involved in neuroendocrine stress response; in pancreatic islets, it may produce a glucose-sensitive effect and decrease insulin levels required to control hyperglycemia [59]. Fibroblast growth factor 2, being involved in cell growth, angiogenesis, atherogenesis, wound healing and other processes, is implicated in the development of diabetic nephropathy [60], diabetic retinopathy [61], diabetic neuropathy [62], and coronary artery disease [63].

For the networks of hypoglycemia and vascular complications, overrepresented Gene Ontology biological processes have been identified using the DAVID web-tool (Table S11). Eleven processes were overrepresented simultaneously for the gene sets of all considered conditions (Table 4). Among these processes, there were those involved in glucose homeostasis, regulation of nitric oxide biosynthesis, muscle cell proliferation, DNA replication and apoptosis, regulation of protein kinase B signaling, ERK1 and ERK2 cascade, and others.

Thus, the comparative analysis of the gene networks of hypoglycemia and diabetic vascular complications indicates common molecular and cellular mechanisms underlying these disorders. The deteriorating effect of hypoglycemia in diabetic vascular disease can be mediated through a wide range of genes encoding hormones, receptors, cytokines, growth factors, and some other proteins that modulate not only glucose homeostasis but also the cell cycle, proliferation and intracellular signaling pathways.

### 2.3. Comparative Analysis of the Gene Networks of Hypoglycemia, Cognitive Decline and AD

At the next step, we matched the gene network of hypoglycemia with the network of cognitive decline and AD. The gene network of cognitive decline is presented in Table S12. It contains 302 genes/proteins, of which 22 were also participating in hypoglycemia gene network. Among the mutual genes, we have identified those encoding insulin (*INS*), islet amyloid polypeptide (*IAPP*), somatostatin (*SST*), leptin (*LEP*), adiponectin (*ADIPOQ*), erythropoietin (*EPO*), interleukin-6 (*IL6*), tumor necrosis factor α (*TNF*), AKT serine/threonine kinase 1 (*AKT1*) and C-C motif chemokine ligand 2 (*CCL2*), insulin-like growth factor 1 (*IGF1*), insulin-like growth factor-binding protein 3 (*IGFBP3*), epidermal growth factor receptor (*EGFR*), adenylate cyclase-activating polypeptide 1 (*ADCYAP1*), albumin (*ALB*), angiotensin I converting enzyme (*ACE*), aquaporin 4 (*AQP4*), C-reactive protein (*CRP*), prion protein (*PRNP*), serpin family A member 1 (*SERPINA1*), serpin family E member 1 (*SERPINE1*), and sirtuin 1 (*SIRT1*). According to the hypergeometric distribution, the genes associated with hypoglycemia were overrepresented in the cognitive decline network (*p*-value 10^−15^).

A growing body of evidence indicates a link between impaired glucose metabolism in the central nervous system and AD [17,64,65,66]. It was shown that reduced glucose availability in the brain directly triggers behavioral deficits by promoting the development of tau neuropathology and synaptic dysfunction [17,18]. Therefore, in this work, we have reconstructed a gene network of AD and matched it with that of hypoglycemia. The network of AD included 1622 genes/proteins (Table S13). Of these molecules, 77 were also involved in the network of hypoglycemia and 22 molecules were associated with hypoglycemia, cognitive decline and AD. It should be noted that genes/proteins related to hypoglycemia were significantly overrepresented in the AD network (*p*-value 10^−45^).

Analysis of the overrepresentation of GO processes associated with AD network (Table S11) revealed the same biological processes that have been identified for hypoglycemia, cardiovascular disease and microvascular diabetic complications (Table 4). In addition, the negative regulation of neuron death was recognized among the top processes related to the AD network. Therefore, it can be assumed that hypoglycemia triggers a number of molecular and cellular events that are universal for vascular and neurological complications.

It was interesting to match the relations of hypoglycemia and AD with associated genes (Table 5, Table S14). Genetic variations that modulate risk of both disorders were found in *ADRB2* [67,68], and *PSMB9* [69,70]. Mutations of *HK1, IGF1R, INSR, NR3C1, SIRT6,* and *WWOX* were associated with hypoglycemia [71,72,73,74,75,76] and these genes were found to be down-regulated in AD [77,78,79,80,81,82]. Eleven genes (*CCL2, CD40, CDKN1A, CYP3A4, FOS, HCRT, IGFBP2, IL6, PARK7, SERPINA1,* and *VEGFA*) were up-regulated by hypoglycemia and were found to be also up-regulated in AD. Another group of genes (*MAP2, METAP2, NFE2L2, SELP, SST,* and *VEGFA*) was down-regulated by hypoglycemia and also down-regulated in AD.

A number of analyzed genes were suggested as potential targets for AD treatment, including *ADIPOQ* [83], *AQP4* [84], *CCL2* [85], *CRP* [86], *EIF2AK3* [87], *EPO* [88], *IGF1* [89], *IGF1R* [90,91], *IGF2* [92,93], *IGFBP2* [94], *IL6* [95], *NFE2L2* [96,97], *NOS1* [98], *NPY* [99,100], *PARK7* [101], *SERPINE1* [102], *SIRT1* [103], *SIRT6* [81,104], *SLC2A1* [105,106], *TNF* [107,108], *VEGF* and *VEGFR* [109]. The role of these molecules in hypoglycemia-induced brain dysfunction needs further research.

Thus, the obtained results demonstrate significant similarity in the gene networks of hypoglycemia, cardiovascular disease, diabetic microvascular complications and AD. This is consistent with clinical evidence that cognitive dysfunction is associated with severe hypoglycemia and the presence of micro- and/or macrovascular diseases in subjects with diabetes [110]. The revealed universality of molecular events and biological processes in hypoglycemia, cardiovascular diseases and AD contributes to a further understanding of the mechanisms of comorbidity in diabetes.

### 2.4. Study Limitations

Our study is not without limitations. As the ANDSystem utilizes an automatic text mining-based approach for network reconstruction, we cannot exclude that some relevant information has been missed. The study is a hypothesis-generating one. The role of identified genes/proteins, as well as biological processes, in hypoglycemia and associated events, needs further experimental testing.

## 3. Materials and Methods

### 3.1. The ANDSystem Tool and Network Analysis

The reconstruction of gene networks was performed using the ANDSystem, version: 20.0413b646_2021 (ICG SB RAS, Novosibirsk, Russia). The ANDSystem is available online: http://www-bionet.sscc.ru/and/cell/ (accessed on 8 September 2021). The main modules of ANDSystem are the knowledge extraction module, the ANDCell knowledge base and the user interface ANDVisio. The knowledge extraction module is based on text-mining technology utilizing the dictionaries of object names and semantic templates. The preparation of dictionaries is based on the automatic extraction of names and synonyms of biological objects from external databases and the texts of scientific publications. The semantic templates are the structured records listing the object types, dictionaries, regular expressions for text analysis and descriptions of the interaction semantics. As a result of the triggering of a linguistic template, interactions between objects from dictionaries are revealed. Linguistic templates generalize interactions by 24 types (for example, expression regulation, protein-protein interaction, association, etc.), and also define the organism in which this interaction is found. The knowledge extraction module allows the filling of the ANDCell knowledge base. It is a prebuild knowledge base which contains information about more than 20 million interactions between biological objects. The update of the information stored in the ANDCell is performed annually. Both the knowledge extraction module and the ANDCell knowledge base are located on a server. The ANDVisio is a client module allowing the user to query the ANDCell knowledge base. Based on the user queries the molecular-genetic networks could be reconstructed, analyzed and visualized as bipartite graphs. Biological objects are shown as nodes and interactions between them are represented as edges of the graph. The ANDSystem could be used for building in an automatic manner the associative molecular (gene) networks describing phenotypes, diseases and biological processes important for bio-medical tasks [22,23,24].

As the ANDVisio allows to analyze the molecular-genetic networks it was applied to find the node connectivity and betweenness centrality coefficients of nodes in the hypoglycemia gene network. These parameters were calculated with function “Statistics” of the “Analysis” section of ANDVisio. The cross-talk specificity (CTS) values were calculated by ANDVisio function “Intelligent Filtration.” CTS was calculated according to the formula: CTS=K_i_/M_i_, where K_i_ is a number of links that the i gene has in the analyzed gene network; M_i_ is a number of links that the i gene has in the global human gene network of ANDSystem [22,23,24].

### 3.2. The Gene Set Enrichment Analysis

The gene set enrichment analysis is broadly used to identify groups of genes that are over-/under-represented in a large gene set and that can possibly be associated with studied conditions based on statistical approaches, for example, using the hypergeometric distribution.

The gene set enrichment analysis web-tool DAVID (Available online: https://david.ncifcrf.gov/home.jsp (accessed on 31 August 2021)), version 6.8 (LHRI, Frederick, MD, USA) [29] was used to find the overrepresented Gene Ontology biological processes. The parameters were set as follows: organism, “*Homo sapiens*”; Gene_Ontology,“GOTERM_BP_DIRECT.” The statistically significant enrichment of a Gene Ontology biological process was considered when the *p*-values with Bonferroni correction were lower than 0.01.

The enrichment analysis of anatomical structures mapped to genes by expression patterns was performed by Bgee (Available online: https://bgee.org/ (accessed on 27 April 2021)) function TopAnat [30].

The assessment of the overrepresentation of hypoglycemia genes in the networks of cardiovascular disease, diabetic nephropathy, diabetic retinopathy, diabetic neuropathy, cognitive decline and AD was performed according to the hypergeometric distribution by the “hypergeom.sf” function of the “scipy” library of the Python programming language [111].

### 3.3. The Databases AmiGO2 and GeneCards

The GeneCards^®^: The Human Gene Database (Available online: https://www.genecards.org/ (accessed on 16 September 2021)) stores information on gene molecular function. It was queried to check the molecular function of identified genes associated with hypoglycemia.

The AmiGO2 database (Available online: http://geneontology.org/ (accessed on 4 September 2021)) [112] is a web-based tool for searching and browsing the Gene Ontology which is the world’s largest knowledge base containing the information on gene functions. The AmiGO2 database was used to find the genes associated with microvascular endothelial cells.

### 3.4. Manual Classification of Links between Genes and Hypoglycemia

Table 2 was built based on information about relations between hypoglycemia and genes automatically extracted from PubMed publications by ANDSystem. The extracted sentences presented in Table S1 were manually analyzed and the links between genes and hypoglycemia were classified in 6 groups: “Gene expression is up-regulated by hypoglycemia,” “Gene expression is down-regulated by hypoglycemia,” “Molecules with hypoglycemic or antihyperglycemic activity,” “Protective effect against hypoglycemia and/or response to hypoglycemia,” “SNPs associated with the risk of hypoglycemia,” and “Other links.”

### 3.5. The Venn Diagram

The Venn diagram demonstrating the interactions of hypoglycemia-related genes from the gene networks of cardiovascular disease, diabetic nephropathy, diabetic retinopathy, diabetic neuropathy, and Alzheimer’s disease was made by the “Bioinformatics & Evolutionary Genomics” resource available online: http://bioinformatics.psb.ugent.be/webtools/Venn/ (accessed on 7 September 2021).

## 4. Conclusions

Hypoglycemia is a trigger for a number of complications and comorbidities in diabetes, including cardiovascular events, microvascular diabetic complications, cognitive dysfunction, and AD. In this work, we reconstructed and matched to each other the gene networks of hypoglycemia and the above-mentioned disorders using the ANDSystem that operates text-mining technology.

There were 141 genes/proteins in the hypoglycemia-associated network. Among them, *INS, IL6, LEP, TNF, IL1B, EGFR,* and *FOS* were the principal central hubs, meanwhile, *GPR142, MBOAT4, SLC5A4, IGFBP6, PPY, G6PC1, SLC2A2, GYS2, GCGR* and *AQP7* were the most specific, according to the CTS criterion. The enrichment analysis of GO biological processes showed that regulation of insulin secretion, glucose homeostasis, apoptosis, nitric oxide biosynthesis and cell signaling are significantly enriched for hypoglycemia. The anatomical structures that are overrepresented among those associated with hypoglycemia genes are the central nervous system, muscles, aorta, connective tissue, and others.

In the next step, we built the gene networks of diabetic complications and comorbidities for which hypoglycemia is considered a trigger. A step-by-step comparison of the hypoglycemic gene network with that for cardiovascular diseases, diabetic retinopathy, diabetic nephropathy, diabetic neuropathy, cognitive decline and AD showed that hypoglycemia-related genes are overrepresented for all hypoglycemia-triggered conditions according to the hypergeometric distribution. It was suggested that 14 genes (*ADIPOQ, CRP, EDN1, EPO, GLP1R, IGF1, IL1B, IL6, INS, INSR, NFE2L2, NPY, TNF,* and *VEGFA)* can significantly contribute to the development of hypoglycemia comorbidities. It turned out that genes associated with hypoglycemia, macro- and microvascular diabetes complications and Alzheimer’s disease are involved in nitric oxide biosynthesis, glucose homeostasis, ERK1 and ERK2 cascade, smooth muscle cell proliferation, and some others. In AD, hypoglycemia also regulates the neuron death process. Among the genes associated with both AD and hypoglycemia, we have identified those that are promising for further study as drug targets (*CCL2, CD40, CDKN1A, CYP3A4, FOS, HCRT, IGFBP2, IL6, MAP2, METAP2, NFE2L2, PARK7, SELP, SST, VEGFA* and others).

The obtained results expand the understanding of the molecular mechanisms of the deteriorating effect of hypoglycemia on the targeted organs in diabetes. Influencing the expression of many genes and intensity of physiological processes, hypoglycemia can play an important role in the promotion of diabetes-associated vascular disease and cognitive dysfunction.

## Figures and Tables

**Table 1:** Molecules of the hypoglycemia network.

Group of Molecules	Genes
Hormones	*ADIPOQ, AVP, EPO, GCG, GH1, GIP, IAPP, INS, LEP, PRL, PTH, REN, SCT,* and *SST*
Cytokines and growth factors	*ANGPTL4, CCL2, CSF3, EDN1, FGF2, FGF21, IGF1, IGF2, IL1B, IL6, TNF,* and *VEGFA*
Receptors	*ADRB2, ADRB3, AGTR2, CD36, CD40, EGFR, GCGR, GHRHR, GLP1R, GPR142, IGF1R, IGF2R, INSR, LEPR, MC2R, NOTCH1, NR3C1, SORCS1,* and *SSTR2*
Enzymes	*ACE, AKT1, AKT2, CHAT, CPT1A, CYP2C9, CYP3A4, DNMT1, DPP4, EIF2AK3, FBP1, G6PC1, GCK, GPD1, GPD2, GPX3, GSR, GYS2, H6PD, HK1, MAP4K2, MBOAT4, METAP2, NAMPT, NOS1, PARK7, PDK4, PGM1, SERPINA1, SIAH2, SIRT1, SIRT6, SOD2, TGM1, TIGAR, UQCRC2, VHL,* and *WWOX*
Transporters	*ABCC8, AQP4, AQP7, CACNA1C, KCNJ11, KCNH2, MPC2, RAMP1, SLC5A1, SLC5A2, SLC5A4, SLC16A1, SLC2A1, SLC2A2, SLC22A1, SLC30A8, SLC25A20, SLC30A10,* and *UCP2*
Transcription factors	*ARNTL, DDIT3, FOS, FOXO1, HNF1A, HNF4A, NFE2L2, NR3C1, PPARA, PROP1, SOX17,* and *TCF7L2*
Neuropeptides	*ADCYAP1, CHGA, GRP, HCRT, NPY, PPY,* and *UCN*
Structural proteins	*CLDN5* and *MAP2*
Other proteins	*ALB, CDKN1A, CISD1, CRP, IGFBP1, IGFBP2, IGFBP3, IGFBP6, NRP1, PRNP, PSMB9, PSMG1, SELE, SELP, SERPINE1, TRAF6,* and *VAMP8*
MicroRNAs	*MIR155* and *MIR410*

**Table 2:** The patterns of the links between identified genes and hypoglycemia.

Link	Genes
Gene expression is up-regulated by hypoglycemia	*ADIPOQ, ALB, ANGPTL4, AQP4, AVP, CCL2, CD40, CDKN1A, CHAT, CHGA, CRP, CYP3A4, DDIT3, EDN1, EIF2AK3, EPO, FOS, GIP, GH1, GPD1, GPX3, GRP, HCRT, IGFBP1, IGFBP2, IL6, LEPR, NOS1, NPY, PARK7, PDK4, PPY, PRL, PRNP, REN, SELE, SERPINE1, SLC2A1, SOD2, SOX17, TIGAR, VEGFA,* and *UCN*
Gene expression is down-regulated by hypoglycemia	*CLDN5, CPT1A, DNMT1, FBP1, GPD2, GSR, MAP2, METAP2, NFE2L2, NRP1, PTH, RAMP1, SELP, SLC25A20, SLC2A1, SST,* and *VEGFA*
Molecules with hypoglycemic or antihyperglycemic activity	*CSF3, GIP, GLP1R, IGF1, IGF2, IGF2R, IGFBP6, IL1B, INS, KCNH2, MIR155, NOTCH1, SCT, SLC16A1, SSTR2,* and *TRAF6*
Protective effect against hypoglycemia and/or response to hypoglycemia	*ADRB3, CD36, FOXO1, GCG, GCGR, DPP4, GH1, IAPP, IGFBP3, KCNH2, LEP, MBOAT4, MIR410, MPC2, PPARA, PRL, SLC2A2, SOD2, TNF, VHL, VAMP8,* and *UCN*
SNPs associated with the risk of hypoglycemia	*ABCC8, ACE, ADRB2, AGTR2, AKT2, AQP7, CACNA1C, CYP2C9, G6PC, GCK, GHRHR, GYS2, HK1, HNF1A, HNF4A, IGF1R, INSR, KCNJ11, MAP4K2, MC2R, NR3C1, PGM1, PROP1, PSMB9, SERPINA1, SIRT6, SLC22A1, SLC2A2, SORCS1, TCF7L2, TGM1, UCP2, UQCRC2,* and *WWOX*
Other links	*ADCYAP1, AKT1, ARNTL, CISD1, EGFR, FGF2, FGF21, GPR142, H6PD, NAMPT, PSMG1, SIAH2, SIRT1, SLC2A2, SLC30A10, SLC30A8, SLC5A1, SLC5A2,* and *SLC5A4*

**Table 3:** Top 10 GO biological processes most overrepresented for hypoglycemia-related genes.

Gene Ontology Biological Process	Genes	*p*-Values with Bonferroni Correction
GO:0050796~regulation of insulin secretion	*ABCC8, ARNTL, CACNA1C, CPT1A, GCG, GCK, GIP, GLP1R, HNF1A, HNF4A, IL1B, KCNJ11, LEP, SLC16A1, SLC2A1, SLC2A2, TNF*	2.66 × 10^−16^
GO:0042593~glucose homeostasis	*ADIPOQ, AKT1, G6PC1, GCG, GCGR, GCK, HNF1A, HNF4A, IL6, INS, INSR, LEP, LEPR, PDK4, SIRT6, SLC16A1, SLC30A8, TCF7L2*	2.16 × 10^−13^
GO:0045944~positive regulation of transcription from RNA polymerase II promoter	*ADCYAP1, ADRB2, AKT1, ARNTL, CD40, CSF3, DDIT3, EDN1, EGFR, FGF2, FOS, FOXO1, HNF1A, HNF4A, IGF1, IL1B, IL6, NAMPT, NFE2L2, NOS1, NOTCH1, NR3C1, PARK7, PPARA, PROP1, PTH, SERPINE1, SIRT1, SOX17, TCF7L2, TNF, TRAF6, UCN, VEGFA, WWOX*	8.36 × 10^−10^
GO:1901215~negative regulation of neuron death	*AKT1, CHGA, CSF3, EPO, FGF21, IL6, PARK7, PPARA, SIRT1, TIGAR, UCN*	1.35 × 10^−09^
GO:0015758~glucose transport	*AKT1, EDN1, G6PC1, GCK, GH1, HK1, INS, SLC2A1, SLC2A2, SLC5A1*	8.35 × 10^−09^
GO:0006006~glucose metabolic process	*ADIPOQ, AKT1, AKT2, CPT1A, H6PD, IGF2, INS, KCNJ11, LEP, PDK4, PGM1, TNF*	1.30 × 10^−08^
GO:0045725~positive regulation of glycogen biosynthetic process	*AKT1, AKT2, GCK, IGF1, IGF2, INS, INSR, PTH*	2.58 × 10^−08^
GO:0043066~negative regulation of apoptotic process	*AKT1, ALB, ANGPTL4, AVP, CDKN1A, EGFR, FOXO1, GCG, IGF1, IGF1R, IL6, LEP, PARK7, PRNP, PROP1, SIAH2, SIRT1, SOD2, TRAF6, UCN, UCP2, VEGFA, VHL*	3.92 × 10^−08^
GO:0045429~positive regulation of nitric oxide biosynthetic process	*AGTR2, AKT1, EDN1, EGFR, IL1B, IL6, INS, INSR, SOD2, TNF*	1.14 × 10^−07^
GO:0046326~positive regulation of glucose import	*ADIPOQ, AKT1, AKT2, FGF21, IGF1, INS, INSR, NFE2L2, PTH*	1.66 × 10^−07^

**Table 4:** Gene ontology biological processes that are overrepresented simultaneously for the gene sets associated with hypoglycemia and cardiovascular disease, diabetic neuropathy, diabetic retinopathy, diabetic nephropathy, and AD.

Gene Ontology Biological Process	Enrichment Significance, *p*-Value with Bonferroni Correction				
	Cardiovascular Disease	Diabetic Neuropathy	Diabetic Retinopathy	Diabetic Nephropathy	AD
GO:0045429~positive regulation of nitric oxide biosynthetic process	2.05 × 10^−08^	1.29 × 10^−06^	3.63 × 10^−08^	3.29 × 10^−09^	2.21 × 10^−08^
GO:0008284~positive regulation of cell proliferation	1.13 × 10^−08^	1.49 × 10^−06^	4.81 × 10^−07^	5.07 × 10^−10^	2.77 × 10^−08^
GO:0045944~positive regulation of transcription from RNA polymerase II promoter	2.38 × 10^−06^	6.50 × 10^−05^	8.74 × 10^−09^	1.49 × 10^−10^	6.93 × 10^−12^
GO:0051897~positive regulation of protein kinase B signaling	1.02 × 10^−04^	3.99 × 10^−05^	4.67 × 10^−06^	2.32 × 10^−08^	5.76 × 10^−09^
GO:0046326~positive regulation of glucose import	1.67 × 10^−07^	3.65 × 10^−05^	2.74 × 10^−07^	1.37 × 10^−06^	3.24 × 10^−04^
GO:0045740~positive regulation of DNA replication	9.77 × 10^−05^	1.47 × 10^−04^	1.49 × 10^−04^	5.77 × 10^−04^	4.78 × 10^−05^
GO:0048661~positive regulation of smooth muscle cell proliferation	6.05 × 10^−04^	6.33 × 10^−04^	2.17 × 10^−05^	5.54 × 10^−08^	1.36 × 10^−05^
GO:0050731~positive regulation of peptidyl-tyrosine phosphorylation	8.86 × 10^−05^	4.33 × 10^−07^	0.004386	2.44 × 10^−05^	4.72 × 10^−06^
GO:0045840~positive regulation of mitotic nuclear division	7.25 × 10^−04^	2.00 × 10^−05^	0.001034	0.003167	1.52 × 10^−04^
GO:0042593~glucose homeostasis	3.09 × 10^−04^	0.005116	1.71 × 10^−05^	1.26 × 10^−07^	1.04 × 10^−06^
GO:0070374~positive regulation of ERK1 and ERK2 cascade	0.007662	9.15 × 10^−07^	1.72 × 10^−06^	3.86 × 10^−08^	0.001765

**Table 5:** Combined analysis of the genes related to hypoglycemia and AD.

	AD	Genes Up-Regulated by AD	Genes Down- Regulated by AD	Genes with SNPs Increasing AD Risk	Genes with Other Relations with AD
Hypoglycemia					
**Genes up-regulated by hypoglycemia**		*CCL2, CD40, CDKN1A, CYP3A4, FOS, HCRT, IGFBP2, IL6, PARK7, SERPINA1, VEGFA*	*AVP, EDN1, LEPR, SLC2A1, VEGFA*	*CHAT, NPY, PRNP*	*ADIPOQ, ALB, AQP4, CHGA, CRP, DDIT3, EIF2AK3, EPO, GH1, GIP, NOS1, SERPINE1, SOD2*
**Genes down-regulated by hypoglycemia**		*VEGFA*	*MAP2, METAP2, NFE2L2, SELP, SST, VEGFA*	*DNMT1*	
**Genes with SNPs increasing hypoglycemia risk**		*CYP2C9*	*HK1, IGF1R, INSR, NR3C1, SIRT6, WWOX*	*ADRB2, PSMB9*	*ACE, IGF2R, PGM1, SORCS1, TCF7L2*
**Genes with other relations with hypoglycemia**		*IGFBP3, IGF2, MIR155, NOTCH1, TNF*	*NAMPT, SIRT1, SLC30A10, SSTR2*	*AKT1, ARNTL, CD36, IL1B, PPARA*	*ADCYAP1, CSF3, EGFR, FGF2, FOXO1, GLP1R, IAPP, IGF1, INS, KCNH2, LEP, SLC16A1*

## Data Availability

The data supported reported results are available in Supplementary Materials to this article.

## Supplementary Materials

The following are available online at www.mdpi.com/article/10.3390/ijms222212419/s1.

## Abbreviations

AD	Alzheimer’s disease
CTS	cross-talk specificity
ERK	extracellular signal-regulated kinase
GO	Gene Ontology
SNP	single nucleotide polymorphism
